# Co-Training for Unsupervised Domain Adaptation of Semantic Segmentation Models

**DOI:** 10.3390/s23020621

**Published:** 2023-01-05

**Authors:** Jose L. Gómez, Gabriel Villalonga, Antonio M. López

**Affiliations:** 1 Computer Vision Center (CVC), Universitat Autònoma de Barcelona (UAB), 08193 Bellaterra, Spain; 2 Computer Science Department, Universitat Autònoma de Barcelona (UAB), 08193 Bellaterra, Spain

**Keywords:** domain adaptation, semi-supervised learning, semantic segmentation, autonomous driving

## Abstract

Semantic image segmentation is a core task for autonomous driving, which is performed by deep models. Since training these models draws to a curse of human-based image labeling, the use of synthetic images with automatically generated labels together with unlabeled real-world images is a promising alternative. This implies addressing an unsupervised domain adaptation (UDA) problem. In this paper, we propose a new *co-training* procedure for synth-to-real UDA of semantic segmentation models. It performs iterations where the (unlabeled) real-world training images are labeled by intermediate deep models trained with both the (labeled) synthetic images and the real-world ones labeled in previous iterations. More specifically, a self-training stage provides two domain-adapted models and a model collaboration loop allows the mutual improvement of these two models. The final semantic segmentation labels (pseudo-labels) for the real-world images are provided by these two models. The overall procedure treats the deep models as black boxes and drives their collaboration at the level of pseudo-labeled target images, i.e., neither modifying loss functions is required, nor explicit feature alignment. We test our proposal on standard synthetic and real-world datasets for onboard semantic segmentation. Our procedure shows improvements ranging from approximately 13 to 31 mIoU points over baselines.

## 1. Introduction

Semantic image segmentation is a central and challenging task in autonomous driving, as it involves predicting a class label (e.g., Road, Pedestrian, Vehicle, etc) per pixel in outdoor images. Therefore, non-surprisingly, the development of deep models for semantic segmentation has received a great deal of interest since deep learning is the core for solving computer vision tasks [1,2,3,4,5,6,7]. In this paper, we do not aim at proposing a new deep model architecture for onboard semantic segmentation, but our focus is on the training process of semantic segmentation models. More specifically, we explore the setting where such models must perform in real-world images, while for training them we have access to automatically generated synthetic images with semantic labels together with unlabeled real-world images. It is well-known that training deep models on synthetic images for performing on real-world ones requires domain adaptation [8,9], which must be unsupervised if we have no labels from real-world images [10]. Thus, this paper falls into the realm of unsupervised domain adaptation (UDA) for semantic segmentation [11,12,13,14,15,16,17,18,19,20,21,22], i.e., in contrast to assuming access to labels from the target domain [23,24]. Note that the great relevance of UDA in this context comes from the fact that, until now, pixel-level semantic image segmentation labels are obtained by cumbersome and error-prone manual work. In fact, this is the reason why the use of synthetic datasets [25,26,27] arouses great interest.

In this paper, we address synth-to-real UDA following a co-training pattern [28], which is a type of semi-supervised learning (SSL) [29,30] approach. Essentially, canonical co-training consists in training two models in a collaborative manner when only a few labeled data are available but we can access a relatively large amount of unlabeled data. In the canonical co-training pattern, the domain shift between labeled and unlabeled data is not present. However, UDA can be instantiated in this paradigm.

**Figure 1:**
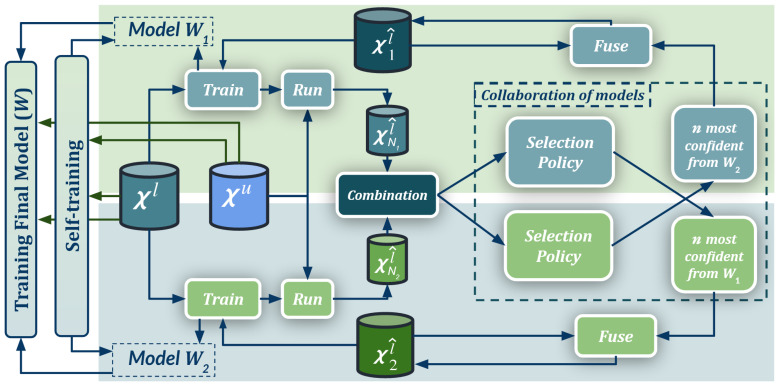
Co-training procedure for UDA. $X^{l}$ is a set of labeled synthetic images, $X^{u}$ a set of unlabeled real-world images, and $X_{x}^{\hat{l}}$ is the set *x* of real-world pseudo-labeled images (automatically generated). Our self-training stage provides two initial domain-adapted models ($W_{1},W_{2}$), which are further trained collaboratively by exchanging pseudo-labeled images. Thus, this procedure treats the deep models as black boxes and drives their collaboration at the level of pseudo-labeled target images, i.e., neither modifying loss functions is required, nor explicit feature alignment. See details in Section 3 and Algorithms 1–3.

In previous works, we successfully applied a co-training pattern under the synth-to-real UDA setting for deep object detection [31,32]. This encourages us to address the challenging problem of semantic segmentation under the same setting by proposing a new co-training procedure, which is summarized in Figure 1. It consists of a self-training stage, which provides two domain-adapted models, and a model collaboration loop for the mutual improvement of these two models. These models are then used to provide the final semantic segmentation labels (pseudo-labels) for the real-world images. In contrast to previous related works, the overall procedure treats the deep models as black boxes and drives their collaboration only at the level of pseudo-labeled target images, i.e., neither modifying loss functions is required, nor explicit feature alignment. We test our proposal on synthetic (GTAV [26], Synscapes [27], SYNTHIA [25]) and real-world datasets (Cityscapes [33], BDD100K [34], Mapillary Vistas [35]) which have become standard for researching on on-board semantic segmentation. Our procedure shows improvements ranging from approximately 13 to 31 mean intersection-over-union (mIoU) points over baselines, being less than 10 mIoU points below upper bounds. Moreover, to the best of our knowledge, we are the first to report synth-to-real UDA results for semantic segmentation in BDD100K and Mapillary Vistas.

In summary, the main contributions of this paper are:

- The design and implementation of a novel co-training procedure to tackle synth-to-real UDA for semantic segmentation. To the best of our knowledge, it is the first time that the co-training pattern [28] is instantiated for such a purpose.
- This procedure allows for seamlessly leveraging multiple heterogeneous synthetic datasets. In this paper, we show the case of joint use of GTAV and Synscapes datasets.
- This procedure is complementary to image pre-processing techniques such as color space adjustments and learnable image-to-image transformations. In this paper, we use LAB alignment.
- This procedure allows for seamlessly leveraging adaptive confidence thresholding and domain mixing techniques. In this paper, we use thresholding inspired by MPT [12], the ClassMix collage transform [36], and mini-batch domain mixes (which we termed as cool-world a decade ago [37]).
- In contrast to the main trend in the related literature, our proposal is purely data-driven. More specifically, we treat semantic segmentation models as black boxes; thus, our co-training neither requires modifying specific semantic segmentation losses nor performing explicit feature alignment.
- Overall, in public benchmarks, our co-training reaches state-of-the-art accuracy on synth-to-real UDA for semantic segmentation.

Section 2 contextualizes our work. Section 3 details the proposed procedure. Section 4 describes the experimental setup and discusses the obtained results. Section 5 summarizes this work. Finally, Appendix A, Appendix B, Appendix C, provide more details regarding datasets and co-training quantitative and qualitative results.

## 2. Related Works

Li et al. [12] and Wang et al. [17] rely on adversarial alignment to perform UDA. While training a deep model for semantic segmentation, it is performed adversarial image-to-image translation (synth-to-real) together with an adversarial alignment of the model features arising from the *source* (synthetic images) and *target* domains (real-world images). Both steps are alternated as part of an iterative training process. For feature alignment, pseudo-labeling of the target domain images is performed. This involves applying an automatically computed per-class max probability threshold (MPT) to class predictions. Tranheden et al. [21] follow the idea of mixing source and target information (synthetic and real) as training samples [25]. However, target images are used after applying ClassMix [36], i.e., a class-based collage between source and target images. This requires the semantic segmentation ground truth, which for the synthetic images (source) is available while for the real-world ones (target) pseudo-labels are used. Such domain adaptation via cross-domain mixed sampling (DACS) is iterated so that the semantic segmentation model can improve its accuracy by eventually producing better pseudo-labels. Gao et al. [19] not only augment target images with source classes but the way around too. Their dual soft-paste (DSP) is used within a teacher–student framework, where the teacher model generates the pseudo-labels. Zou et al. [11] propose a self-training procedure where per-cycle pseudo-labels are considered by following a self-paced curriculum learning policy. An important step is class-balanced self-training (CBST), which is similar to MPT since a per-class confidence-based selection of pseudo-labels is performed. Spatial priors (SP) based on the source domain (synth) are also used. The authors improved their proposal in [15] by incorporating confidence regularization steps for avoiding error drift in the pseudo-labels.

Chao et al. [18] assumes the existence of a set of semantic segmentation models independently pre-trained according to some UDA technique. Then, the pseudo-label confidences coming from such models are unified, fused, and finally distilled into a student model. Zhang et al. [22] propose a multiple fusion adaptation (MFA) procedure, which integrates online-offline masked pseudo-label fusion, single-model temporal fusion, and cross-model fusion. To obtain the offline pseudo-labels, existing UDA methods must be applied. In particular, the so-called FDA [16] method is used to train two different models which produce two maps of offline pseudo-labels for each target image. The other two models, $m_{1}\;\&\;m_{2}$, are then iteratively trained. Corresponding temporal moving average models, ${\hat{m}}_{1}\;\&\;{\hat{m}}_{2}$, are kept and used to generate the online pseudo-labels. The training total loss seeks consistency between class predictions of each $m_{i}$ and both offline pseudo-labels and class predictions from the corresponding ${\hat{m}}_{i}$. Moreover, consistency between the online pseudo-labels from ${\hat{m}}_{i}$ and the predictions from $m_{j}$, i≠j, is used as a collaboration mechanism between models. Offline and online pseudo-labels are separately masked out by corresponding CBST-inspired procedures. He et al. [20] assumes the existence of different source domains. To reduce the visual gap between each source domain and the target domain there is a first step where their LAB color spaces are aligned. Then, there are as many semantic segmentation models to train as source domains. Model training relies on source labels and target pseudo-labels. The latter are obtained by applying the model to the target domain images and using a CBST-inspired procedure for thresholding the resulting class confidences. The training of each model is performed iteratively so that the relevance of pseudo-labels follows a self-paced curriculum learning. Collaboration between models is also part of the training. In particular, it is encouraged agreement on the confidence of the different models when applied to the same source domain, for all source domains. Qin et al. [14] proposed a procedure consisting of feature alignment based on cycleGAN [38], additional domain alignment via two models whose confidence discrepancies are considered, and a final stage where the confidences of these models are combined to obtain pseudo-labels which are later used to fine-tune the models. Luo et al. [13] focused on the lack of semantic consistency (some classes may not be well aligned between source and target domains, while others can be). Rather than a global adversarial alignment between domains, a per-class adversarial alignment is proposed. Using a common feature extractor, but two classification heads, per-class confidence discrepancies between the heads are used to evaluate class alignment. The classification heads are forced to be different by a cosine distance loss. Combining the confidences of the two classifiers yields the final semantic segmentation prediction. This approach does not benefit from pseudo-labels.

In contrast to these methods, our proposal is purely data-driven in the sense of neither requiring changing the loss function of the selected semantic segmentation model, nor explicit model features alignment of the source and the target domains via loss function, i.e., we treat the semantic segmentation model as a black box. Our UDA is inspired by co-training [28], so we share with some of the reviewed works the benefit of leveraging pseudo-labels. In our proposal, two models collaborate at a pseudo-label level for compensating labeling errors. These two models arise from our previous self-training stage, which shares with previous literature the self-paced learning idea and adaptive thresholding inspired by MPT, as well as pixel-level domain mixes inspired by ClassMix. Our proposal is complementary to image pre-processing techniques such as color space adjustments and learnable image-to-image transformations. In the case of having multiple synthetic domains, we assume they are treated as a single (heterogeneous) source domain, which has been effective in other visual tasks [39].

## 3. Method

In this section, we explain our data-driven co-training procedure, i.e., the self-training stage, and the model collaboration loop for the mutual improvement of these two models, which we call *co-training loop*. Overall, our proposal works at the pseudo-labeling level, i.e., it does not change the loss function of the semantic segmentation model under training. Global transformations (e.g., color corrections, learnable image-to-image transformations) on either source or target domain images are seen as pre-processing steps. Moreover, in the case of having access to multiple synthetic datasets, whether to use them one at a time or simultaneously is just a matter of the input parameters passed to our co-training procedure.

### 3.1. Self-Training Stage

Algorithm 1 summarizes the self-training stage, which we detail in the following.

**Input & output parameters.** The input $X^{l}$ refers to the set of fully labeled source images; while $X^{u}$ refers to the set of unlabeled target images. In our UDA setting, the source images are synthetic and have automatically generated per-pixel semantic segmentation ground truth (labels), while the target images are acquired with real-world cameras. W refers to the weights of the semantic segmentation model (a CNN) already initialized (randomly or by pre-training on a previous task); while $H_{W}$ are the usual hyper-parameters required for training the model in a supervised manner (e.g., optimization policy, number of iterations, etc). $H_{st}=\left\{T,N,n,K_{m},K_{M},M_{df}\right\}$ consists of parameters specifically required by the proposed self-training. $K_{M}$ is the number of self-training cycles, where we output the model, $W_{K_{M}}$, at the final cycle. $K_{m}$, $K_{m}<K_{M}$, indicates an intermediate cycle from where we also output the corresponding model, $W_{K_{m}}$. *N* is the number of target images used to generate pseudo-labels at each cycle, while *n*, n<N, is the number of pseudo-labeled images to be kept for the next model re-training. $H_{st}$ also contains $T=\{p_{m},p_{M},\Delta p,C_{m},C_{M}\}$, a set of parameters to implement a self-paced curriculum learning policy for obtaining pseudo-labels from model confidences, which is inspired by MPT [12]. Finally, $M_{df}=\left\{p_{MB},p_{CM}\right\}$ consists of parameters to control how source and target images are combined.

**Algorithm 1:** Self-Training Stage.**Input**  : Set of labeled images: $X^{l}$     Set of unlabeled images: $X^{u}$     Net. init. weights & training hyp.-p.: $W,H_{W}$     Self-t. hyp.-p.: $H_{st}=\left\{T,N,n,K_{m},K_{M},M_{df}\right\}$ **Output**: Two refined models: $W_{K_{m}},W_{K_{M}}$               // Initialization             $W_{0}$ ←$\mathrm{BasicModelTraining}(W,H_{W},X^{l})$ $<X^{\hat{l}},k,V_{C_{T}},W>$ ←$<\emptyset ,0,0,W_{0}>$

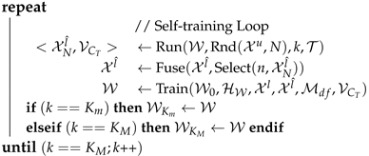


**return**

$W_{K_{m}},W_{K_{M}}$



**Initialization.** We start by training a model, $W_{0}$, on the (labeled) source images, $X^{l}$, according to W and $H_{W}$. At each self-training cycle, $W_{0}$ is used as a pre-trained model.

**Self-training cycles (loop).** Each cycle starts by obtaining a set of pseudo-labeled images, $X_{N}^{\hat{l}}$. For the sake of speed, we do not consider all the images in $X^{u}$ as candidates to obtain pseudo-labels. Instead, *N* images are *selected* from $X^{u}$ and, then, the current model W is applied to them (*run*). Thus, we obtain *N* semantic maps. Each map can be seen as a set of confidence channels, one per class. Thus, for each class, we have *N* confidence maps. Let’s term as $V_{c}$ the vector of confidence values >0 gathered from the *N* confidence maps of class *c*. For each class *c*, a confidence threshold, $C_{T_{c}}$, is set as the value required for having p% values of vector $V_{c}$ over it, where $p=\min(p_{m}+k\Delta p,p_{M})$. Let’s term as $V_{C_{T}}$ the vector of confidence thresholds from all classes. Now, $V_{C_{T}}$ is used to perform per-class thresholding on the *N* semantic segmentation maps, so obtaining the *N* pseudo-labeled images forming $X_{N}^{\hat{l}}$. Note how the use of $p_{m}+k\Delta p$, where *k* is the self-training cycle, acts as a mechanism of *self-paced curriculum learning* on the thresholding process. The maximum percentage, $p_{M}$, allows for preventing noise due to accepting too many per-class pseudo-labels eventually with low confidence. Moreover, for any class *c*, we apply the rule $C_{T_{c}}\leftarrow \max\left(C_{m},\min\left(C_{T_{c}},C_{M}\right)\right)$; where, irrespective of p%, $C_{m}$ prevents from considering not sufficiently confident pseudo-labels, while $C_{M}$ ensures to consider pseudo-labels with a sufficiently high confidence. Then, in order to set the final set of pseudo-labels during each cycle, only *n* of the *N* pseudo-labeled images are *selected*. In this case, an image-level confidence ranking is established by simply averaging the confidences associated to the pseudo-labels of each image. The top-*n* most confident images are considered and *fused* with images labeled in previous cycles. If one of the selected *n* images was already incorporated in previous cycles, we kept the pseudo-labels corresponding to the highest image-level confidence average. The resulting set of pseudo-labeled images is termed as $X^{\hat{l}}$.

Finally, we use the (labeled) source images, $X^{l}$, and the pseudo-labeled target images, $X^{\hat{l}}$, to *train* a new model, W, by fine-tuning $W_{0}$ according to the hyper-parameters $H_{W}$ and $M_{df}$. A parameter we can find in any $H_{W}$ is the number of images per mini-batch, $N_{MB}$. Then, given $M_{df}=\left\{p_{MB},p_{CM}\right\}$, for training W we use $p_{MB}N_{MB}$ images from $X^{\hat{l}}$ and the rest from $X^{l}$. In fact, the former undergoes a ClassMix-inspired collage transform [36]. In particular, we select $p_{MB}N_{MB}$ images from $X^{l}$ and, for each one individually, we gather all the class information (appearance and labels) until considering a $p_{CM}\%$ of classes, going from less to more confident ones, which is possible thanks to $V_{C_{T}}$. This information is pasted at the appearance level (class regions from the source on top of target images) and at the label level (class labels from the source on top of the pseudo-label maps of the target images).

**Algorithm 2:** Collaboration of Models.**Input**   : Sets of pseudo-labeled images: $X_{N_{1}}^{\hat{l}},X_{N_{2}}^{\hat{l}}$     Vectors of per-class conf. thr.: ${V_{C_{T}}}_{1},{V_{C_{T}}}_{2}$     Amount of images to exchange: *n*     Image-level confidence threshold control: λ **Output**: New sets of ps.-lab. images: $X_{N_{1},new}^{\hat{l}},X_{N_{2},new}^{\hat{l}}$ // ClassSort(v) returns the vector of sorted *v* indices after // sorting by *v* values, so that $\Delta_{i}\left[k\right]$ is a class index.          $\Delta_{1}$ ←$\mathrm{ClassSort}({V_{C_{T}}}_{2}-{V_{C_{T}}}_{1})$          $\Delta_{2}$ ←$\mathrm{ClassSort}({V_{C_{T}}}_{1}-{V_{C_{T}}}_{2})$ // ClassImageList(X) returns a vector so that $S_{i}\left[k\right]$ is the // list of images in X containing pseudo-labels of class *k*.          $S_{1}$ ←$\mathrm{ClassImageList}(X_{N_{1}}^{\hat{l}}),$          $S_{2}$ ←$\mathrm{ClassImageList}(X_{N_{2}}^{\hat{l}})$ $X_{N_{1},new}^{\hat{l}},X_{N_{2},new}^{\hat{l}}$ ←∅,∅         $k,N_{c}$ ←0,Num. Classes

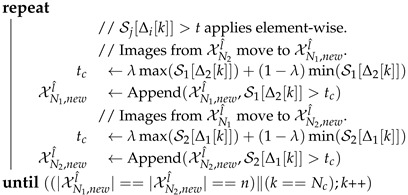


**return**

$X_{N_{1},new}^{\hat{l}},X_{N_{2},new}^{\hat{l}}$



**Algorithm 3:** Co-training Procedure **Uses:** Algorithms 1 & 2.**Input**   : Set of labeled images: $X^{l}$     Set of unlabeled images: $X^{u}$     Net. init. weights & training hyp.-p.: $W,H_{W}$     Self-t. hyp.-p.: $H_{st}=\left\{T,N,n,K_{m},K_{M},M_{df}\right\}$     Co-t. hyp.-p.: $H_{ct}=\left\{K,w,\lambda \right\}$ **Output**: Refined model: W                 // Initialization              $W_{0,1},W_{0,2}$ ←$\mathrm{SelfTraining}(X^{l},X^{u},W,H_{W},H_{st})$ $X_{1}^{\hat{l}},X_{2}^{\hat{l}},k,{V_{C_{T}}}_{1},{V_{C_{T}}}_{2},W_{1},W_{2}$ ←$\emptyset ,\emptyset ,0,0,0,W_{0,1},W_{0,2}$

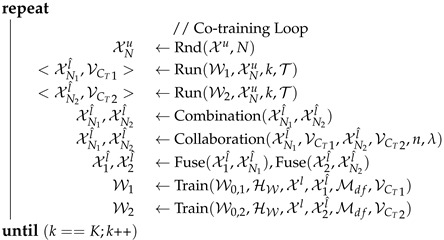

W←$\mathrm{LastTrain}(w,W_{1},W_{2},H_{W},X^{l},X^{u},M_{df})$ **return**W

### 3.2. Co-Training Procedure

Algorithm 3 summarizes the co-training procedure supporting the scheme shown in Figure 1, which is based on the previous self-training stage (Algorithm 1), on combining pseudo-labels, as well as on a model collaboration stage (Algorithm 2). We detail Algorithm 3 in the following.

**Input & output parameters, and Initialization.** Since the co-training procedure includes the self-training stage, we have the input parameters required for Algorithm 1. As additional parameters we have $H_{ct}=\left\{K,w,\lambda \right\}$, where *K* is the maximum number of iterations for mutual model improvement, which we term as *co-training loop*, *w* is just a selector to be used in the last training step (after the co-training loop), and λ is used during pseudo-label exchange between models. The output parameter, W, is the final model. The co-training procedure starts by running the self-training stage.

**Co-training cycles (loop).** Similarly to self-training, a co-training cycle starts by obtaining pseudo-labeled images. In this case two sets, $X_{N_{1}}^{\hat{l}}\&X_{N_{2}}^{\hat{l}}$, are obtained since we *run* two different models, $W_{1}\&W_{2}$. These are applied to the same subset, $X_{N}^{u}$, of *N* unlabeled images *randomly selected* from $X^{u}$. As for self-training, we not only obtain $X_{N_{1}}^{\hat{l}}\&X_{N_{2}}^{\hat{l}}$, but also corresponding vectors of per-class confidence thresholds, ${V_{C_{T}}}_{1}\&{V_{C_{T}}}_{2}$. Since $X_{N_{1}}^{\hat{l}}\&X_{N_{2}}^{\hat{l}}$ come from the same $X_{N}^{u}$ but result from different models, we can perform a simple step of pseudo-label *combination*. In particular, for each image in $X_{N_{i}}^{\hat{l}}$, if a pixel has the *void* class as pseudo-label, then, if the pseudo-label for the same pixel of the corresponding image in $X_{N_{j}}^{\hat{l}}$ is not *void*, we adopt such pseudo-label, i∈{1,2},j∈{1,2},i≠j. This step reduces the amount of non-labeled pixels while keeping pseudo-labeling differences between $X_{N_{1}}^{\hat{l}}\&X_{N_{2}}^{\hat{l}}$ at non-void pseudo-labels.

Note that co-training strategies assume that the models under collaboration perform in a complementary manner. Therefore, after this basic combination of pseudo-labels, a more elaborated *collaboration* stage is applied, which is described in Algorithm 2. Essentially, *n* pseudo-labeled images from $X_{N_{i}}^{\hat{l}}$ will form the new $X_{N_{j}}^{\hat{l}}$ after such collaboration, i∈{1,2},j∈{1,2},i≠j. Thus, along the co-training cycle, pseudo-labeled images arising from $W_{i}$ will be used to retain $W_{j}$. In particular, visiting first those images containing classes for which $X_{N_{i}}^{\hat{l}}$ is more confident than $X_{N_{j}}^{\hat{l}}$, sufficiently high confident images in $X_{N_{i}}^{\hat{l}}$ are selected for the new $X_{N_{j}}^{\hat{l}}$ set, until reaching *n*. The class confidences of $X_{N_{i}}^{\hat{l}}\&X_{N_{j}}^{\hat{l}}$ are given by the respective ${V_{C_{T}}}_{1}\&{V_{C_{T}}}_{2}$, while the confidence of a pseudo-labeled image is determined as the average of the confidences of its pseudo-labels. Being sufficiently high confident means that the average is over a dynamic threshold controlled by the λ parameter.

Once this process is finished, we have two new sets of pseudo-labels, $X_{N_{1}}^{\hat{l}}\&X_{N_{2}}^{\hat{l}}$, which are used separately for finishing the co-training cycle. In particular, each new $X_{i}^{\hat{l}}$ is used as its self-training counterpart (see $X^{\hat{l}}$ in the loop of Algorithm 1), i.e., performing the fusion with the corresponding set of pseudo-labels from previous cycles and fine-tuning of $W_{0,i}$. Finally, once the co-training loops finish, the last train is performed. In this case, the full $X^{u}$ is used to produce pseudo-labels. For this task, we can use an ensemble of $W_{1}$ and $W_{2}$ (e.g., averaging confidences), or any of these two models individually. This option is selected according to the parameter *w*. In this last training, the ClassMix-inspired procedure is not applied, but mixing source and target images at the mini-batch level is still performed according to the value $p_{MB}\in M_{df}$. It is also worth noting that, inside the co-training loop, the two Run() operations can be parallelized, and the two Train() too.

## 4. Experimental Results

### 4.1. Datasets and Evaluation

Our experiments rely on three well-known synthetic datasets used for UDA semantic segmentation as source data, namely, GTAV [26], SYNTHIA [25] and Synscapes [27]. GTAV is composed of 24,904 images with a resolution of 1914×1052 pixels directly obtained from the render engine of the videogame GTA V. Synscapes is composed by 25,000 images with a resolution of 1440×720 pixels of urban scenes, obtained by using a physic-based rendering pipeline. SYNTHIA is composed of 9000 images of urban scenes highly populated, with a resolution of 1280×760 pixels, generated by a videogame-style rendering pipeline based on the Unity3D framework. As real-world datasets (target domain) we rely on Cityscapes [33], BDD100K [34] and Mapillary Vistas [35]. Cityscapes is a popular dataset composed of on-board images acquired at different cities in Germany under clean conditions (e.g., no heavy occlusions or bad weather), it is common practice to use 2975 images for training semantic segmentation models, and 500 images for reporting quantitative results. The latter is known as the validation set. Cityscapes images have a resolution of 2048×1024 pixels. Another dataset is BDD100K, which contains challenging onboard images taken from different vehicles, in different US cities, and under diverse weather conditions. The dataset is divided into 7000 images for training purposes and 1000 for validation. However, a high amount of training images are heavily occluded by the ego vehicle, thus, for our experiments, we rely on an occlusion-free training subset of 1777 images. Nevertheless, we use the official validation set of BDD100K without any image filtering. Image resolution is 1280×720 pixels. Finally, Mapillary Vistas is composed of high-resolution images of street views around the world. These images have a high variation in resolutions and aspect ratios due to the fact that are taken from diverse devices such as smartphones, tablets, professional cameras, etc. For simplicity, we only consider those images with an aspect ratio of 4:3, which, in practice, are more than 75%. Then, we have 14,716 images for training and 1617 for validation.

As is common practice, we evaluate the performance of our system on the validation set of each real-world (target) dataset using the 19 official classes defined for Cityscapes. These 19 classes are common in all the datasets except in SYNTHIA that only contains 16 of these 19 classes, additional dataset-specific classes are ignored for training and evaluation. Note that, although there are semantic labels available for the target datasets, for performing UDA we ignore them at training time, and we use them at validation time. In other words, we only use the semantic labels of the validation sets, with the only purpose of reporting quantitative results. All the synthetic datasets provide semantic labels, since they act as the source domain, we use them. In addition, we note that for our experiments we do not perform any learnable image-to-image transform to align synthetic and real-world domains (like GAN-based ones). However, following [20], we perform synth-to-real LAB space alignment as a pre-processing step.

As is standard, quantitative evaluation relies on PASCAL VOC intersection-over-union metric IoU=TP/(TP+FP+FN) [40], where TP, FP, and FN refer to true positives, false positives, and false negatives, respectively. IoU can be computed per class while using a mean IoU (mIoU) to consider all the classes at once.

### 4.2. Implementation Details

We use the Detectron2 [41] framework and leverage their implementation of DeepLabV3+ for semantic segmentation, with ImageNet weight initialization. We chose the V3+ version of DeepLab instead of the V2 because it provides a configuration that fits well in our 12 GB-memory GPUs, turning out in a ×2 training speed over the V2 configuration and allowing a higher batch size. Other than this, V3+ does not provide accuracy advantages over V2. We will see it when discussing Table 1, where the baselines of V3+ and V2 perform similarly (SYNTHIA case) or V3+ may perform worse (GTAV case). The hyper-parameters used by our co-training procedure are set according to Table 2. Since their meaning is intuitive, we just tested some reasonable values but did not perform a hyperparameter search. As we can see in Table 2 they are pretty similar across datasets. This table does not include the hyper-parameter related to the training of DeepLabV3+, termed as $H_{W}$ in Algorithms 1–3 since they are not specific to our proposal. Thus, we summarize them in the following.

For training the semantic segmentation models, we use SGD optimizer with a starting learning rate of 0.002 and momentum 0.9. We crop the training images to 1024×512 pixels, 816×608, and 1280×720, when we work with Cityscapes, Mapillary Vistas, and BDD100K, respectively. Considering this cropping and our available hardware, we set batch sizes ($N_{MB}$) of four images, four, and two, for these datasets, respectively. Moreover, we perform data augmentation consisting of random zooms and horizontal flips. For computing each source-only baseline model ($W_{0}$ in Algorithm 1) and the final model (returned W in Algorithm 3) we use a two-step learning rate decay of 0.1 at 1/3 and 2/3 of the training iterations. In these cases, the number of iterations is set to 60K when we work with Cityscapes and Mapillary Vistas, and 120 K for BDD100 K to maintain consistency given the mentioned batch sizes. The number of iterations for the self-training stage and the co-training loop is equally set to 8K for Cityscapes and Mapillary Vistas, and 16K for BDD100K.

For training only using GTAV, a class balancing sample policy (CB) is applied. Due to the scarcity of samples from several classes (e.g., bicycle, train, rider, and motorcycle), these are under-represented during training. A simple, yet efficient, method to balance the frequency of samples from these classes is computing individual class frequency in the whole training dataset and applying a higher selection probability for the under-represented classes. The other synthetic datasets in isolation and the combination of GTAV + Synscapes are already well-balanced and we do not need to apply this technique.

### 4.3. Comparison with the State of the Art

In Table 1 we compare our co-training procedure with state-of-the-art methods when using Cityscapes as the target domain. We divide the results into four blocks according to the source images we use: SYNTHIA, GTAV, Synscapes, or GTAV+Synscapes. Most works in the literature present their results only using GTAV or SYNTHIA as source data. We obtain the best results in the SYNTHIA case, with 56 mIoU (19 classes), and for GTAV with 59.5 mIoU. On the other hand, each proposal from the literature uses its own CNN architecture and pre-trained models. Thus, we have added the mIoU score of the baseline that each work uses as starting point to improve according to the corresponding proposed method. Then, we show the difference between the final achieved mIoU score and the baseline one. In Table 1 this corresponds to column Δ(Diff.). Note how our method reaches 20.6 and 31.0 points of mIoU increment on SYNTHIA and GTAV, respectively. The highest for GTAV, and the highest for SYNTHIA on pair with the ProDA proposal. Additionally, for the sake of completeness, we have added the mIoU scores for the 13 classes setting of SYNTHIA since it is also a common practice in the literature. We can see that co-training obtains the best mIoU too. On the other hand, we are mostly interested in the 19-class setting. Using Synscapes as source data we achieved state-of-the-art results in both Δ(Diff.) (13.3 points) and the final mIoU score (58.3). Note that, in this case, our baseline score is similar to the ones reported in previous literature.

By performing a different LAB transform for each synthetic dataset individually, our co-training procedure allows us to join them as if they were one single domain. Thus, we have considered this setting too. Preliminary baseline experiments (i.e., without performing co-training) showed that the combinations GTAV + Synscapes and GTAV + Synscapes + SYNTHIA are the best performings, with a very scarce mIoU difference between them (0.62). Thus, for the shake of bounding the number of experiments, we have chosen GTAV + Synscapes as the only case combining datasets, so also avoiding the problem of the 19 vs. 16 classes discrepancy when SYNTHIA is combined with them. In fact, using GTAV + Synscapes, we reach a Δ(Diff.) of 20.2 points, with a final mIoU of 70.2, which outperforms the second best in 11.2 points, and it clearly improves the mIoU with respect to the use of these synthetic datasets separately (15.6 points comparing to GTAV, 11.9 for Synscapes). Again, in this case, our baseline score is similar to the ones reported in previous literature.

### 4.4. Ablative Study and Qualitative Results

In Table 3 we compare co-training results with corresponding baselines and upper bounds. We also report the results of applying LAB adjustment as only the UDA step, as well as the results from one of the models obtained after our self-training stage (we chose the model from the last cycle). Overall, in all cases, the co-training loop (which completes the co-training procedure) improves the self-training stage, and this stage, in turn, improves over LAB adjustment. Moreover, when combining GTAV + Synscapes we are only 7.95 mIoU points below the upper bound, after improving 20.22 mIoU points over the baseline.

To complement our experimental analysis, we summarize in Table 4 the contribution of the main components of our proposal for the case GTAV + Synscapes → Cityscapes. First, we can see how a proper pre-processing of the data is relevant. In particular, performing synth-to-real LAB space alignment already allows improving 9.31 points of mIoU. This contribution can also be seen in Table 3 and Table 5, where improvements range from 2.59 mIoU points (GTAV+Synscapes→Mapillary Vistas) to 9.34 (GTAV→Cityscapes). This LAB adjustment is a step hardly seen in synth-to-real UDA literature which should not be ignored. Then, back to Table 4, we see that properly combining labeled source images and pseudo-labeled target images (MixBatch) is also relevant since it provides an additional gain of 6.86 points. Note that this MixBatch is basically the *cool world* idea which we can trace back to work of our own lab done before the deep learning era in computer vision [37]. In addition, performing our ClassMix-inspired collage also contributes 1.29 points of mIoU, and the final collaboration of models returns 2.76 additional points of mIoU. Overall, the main components of our synth-to-real UDA procedure contribute 10.91 points of mIoU and LAB alignment 9.31 points. We conclude that all the components of the proposed procedure are relevant.

In order to confirm these positive results, we applied our method to two additional target domains which are relatively challenging, namely, Mapillary Vistas and BDD100K. In fact, up to the best of our knowledge, in the current literature, there are no synth-to-real UDA semantic segmentation results reported for them. Our results can be seen in Table 5, directly focusing on the combination of GTAV + Synscapes as the source domain. In this case, the co-training loop improves less over the intermediate self-training stage. Still, for BDD100K the final mIoU is only 8.22 mIoU points below the upper bound, after improving 24.13 mIoU points the baseline. For Mapillary Vistas our method remains only 9.14 mIoU points below the upper bound and improves 16.11 mIoU points the baseline. To the best of our knowledge, these are state-of-the-art results for BDD100K and Mapillary Vistas when addressing synth-to-real UDA semantic segmentation.

**Figure 2:**
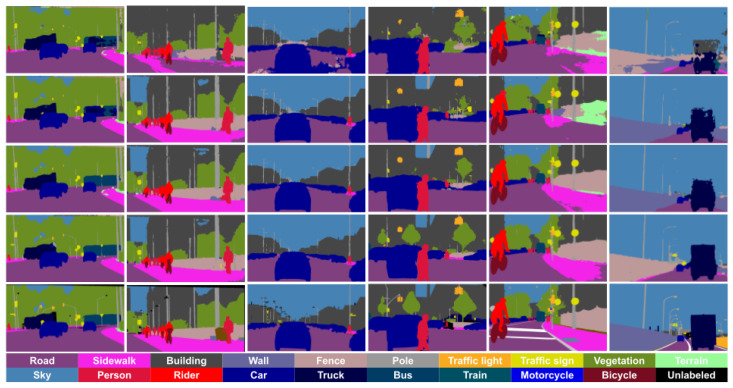
Qualitative results using GTAV + Synscapes as source domain. From (**left**) to (**right**), the two first columns correspond to Cityscapes in the role of the target domain, the next two columns to BDD100K, and the last two to Mapillary Vistas. (**Top**) to (**bottom**) rows correspond to SrcLAB, self-training stage, full co-training procedure, upper bound, and ground truth, respectively.

Figure 2 presents qualitative results of semantic segmentation for the different real-world (target) datasets when using GTAV + Synscapes as the source domain. We observe how the baselines have problems with dynamic objects (e.g., cars, trucks) and some infrastructure classes such as Sidewalk are noisy. The self-training stage mitigates the problems observed in the only-source (with LAB adjustment) results to a large extent. However, we can still observe instabilities in classes such as Truck or Bus, which the co-training loop (full co-training procedure) achieves to address properly. Nevertheless, the co-training procedure is not perfect and several errors are observed in some classes preventing them to reach upper-bound mIoU. In fact, the upper bounds are neither perfect, which is due to the difficulty of performing semantic segmentation in onboard images.

**Figure 3:**
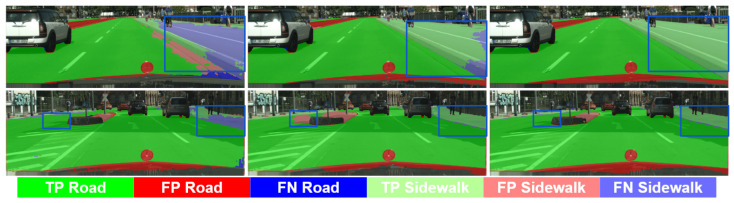
Qualitative results (GTAV + Synscapes → Cityscapes) focusing on TP/FP/FN for road and sidewalk classes. Columns, (**left**) to (**right**): SrcLAB, self-training stage, co-training loop (full co-training procedure). Blue boxes highlight areas of interest.

**Figure 4:**
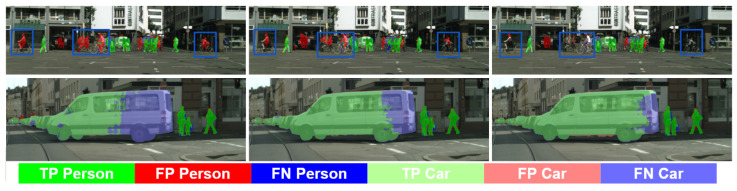
Analogous to Figure 3 for the classes Person and Car.

Figure 3 and Figure 4 exemplify these comments by showing the pseudo-labeling evolution for several classes of special interests such as Road, Sidewalk, Pedestrian, and Car. In Figure 3, we see how the SrcLAB model has particular problems segmenting well the sidewalk, however, the self-training stage resolves most errors although it may introduce new ones (mid-bottom image), while the co-training loop is able to recover from such errors. In Figure 4, we can see (bottom row) how the self-training stage improves the pseudo-labeling of a van, while the co-training loop improves it even more. Analogously, we can see (top row) how self-training helps to alleviate the confusion between Pedestrian and Rider classes, while the co-training loop almost removes all the confusion errors between these two classes.

## 5. Conclusions

In this paper, we have addressed the training of semantic segmentation models under the challenging setting of synth-to-real unsupervised domain adaptation (UDA), i.e., assuming access to a set of synthetic images (source) with automatically generated ground truth together with a set of unlabeled real-world images (target). We have proposed a new co-training procedure combining a self-training stage and a co-training loop where two models arising from the self-training stage collaborate for mutual improvement. The overall procedure treats the deep models as black boxes and drives their collaboration at the level of pseudo-labeled target images, i.e., neither modifying loss functions is required, nor explicit feature alignment. We have tested our proposal on standard synthetic (GTAV, Synscapes, SYNTHIA) and real-world datasets (Cityscapes, BDD100K, Mapillary Vistas). Our co-training shows improvements ranging from approximately 13 to 31 mIoU points over baselines, remaining closely (less than 10 points) to the upper bounds. In fact, up to the best of our knowledge, we are the first to report such results for challenging target domains such as BDD100K and Mapillary Vistas. Moreover, we have shown how the different components of our co-training procedure contribute to improving the final mIoU. Future work, will explore collaboration from additional perception models at the co-training loop, i.e., not necessarily based on semantic segmentation but such collaborations may arise from object detection or monocular depth estimation.

## Figures and Tables

**Table 1:** UDA results. mIoU considers the 19 classes. mIoU* considers 13 classes, which only applies to SYNTHIA, where classes with ’*’ are not considered for global averaging and those with ’-’ scores do not have available samples. Δ(Diff.) refers to the mIoU improvement over the corresponding baseline (i.e., column ’mIoU’—column ’Baseline’). Note also that, following Cityscapes class naming, the class Person refers to pedestrians (i.e., it does not include riders. **Bold** stands for best, and underline for second best. In this table, the target domain is always Cityscapes.

Methods	Road	Sidewalk	Building	Wall *	Fence *	Pole *	Traffic Light	Traffic Sign	Vegetation	Terrain	Sky	Person	Rider	Car	Truck	Bus	Train	Motorbike	Bike	mIoU*	mIoU	Baseline	Δ(Diff.)
SYNTHIA (Source) → Cityscapes																							
AdSegNet [42]	81.7	39.1	78.4	11.1	0.3	25.8	6.8	9.0	79.1	-	80.8	54.8	21.0	66.8	-	34.7	-	13.8	29.9	45.8	39.6	33.5	+6.1
IntraDA [43]	84.3	37.7	79.5	5.3	0.4	24.9	9.2	8.4	80.0	-	84.1	57.2	23.0	78.0	-	38.1	-	20.3	36.5	48.9	41.7	33.5	+8.2
CBST [11]	68.0	29.9	76.3	10.8	1.4	33.9	22.8	29.5	77.6	-	78.3	60.6	28.3	81.6	-	23.5	-	18.8	39.8	48.9	42.6	29.2	+13.4
CRST [15]	67.7	32.2	73.9	10.7	1.6	37.4	22.2	31.2	80.8	-	80.5	60.8	29.1	82.8	-	25.0	-	19.4	45.3	50.1	43.8	34.9	+8.9
DACS [21]	80.5	25.1	81.9	21.4	2.8	37.2	22.6	23.9	83.6	-	90.7	67.6	38.3	82.9	-	38.9	-	28.4	47.5	54.8	48.3	29.4	+18.9
DSP [19]	86.4	42.0	82.0	2.1	1.8	34.0	31.6	33.2	87.2	-	88.5	64.1	31.9	83.8	-	65.4	-	28.8	54.0	59.9	51.0	33.5	+17.5
MFA [22]	81.8	40.2	85.3	-	-	-	38.0	33.9	82.3	-	82.0	73.7	41.1	87.8	-	56.6	-	46.3	63.8	62.5	-	-	-
RED [18]	88.6	46.6	83.7	22.6	4.1	35.0	35.9	36.1	82.8	-	81.3	61.6	32.1	87.9	-	52.7	-	31.9	57.6	59.9	52.5	35.3	+17.2
ProDA [44]	87.8	45.7	84.6	37.1	0.6	44.0	54.6	37.0	88.1	-	84.4	74.2	24.3	88.2	-	51.1	-	40.5	45.6	62.0	55.5	34.9	**+20.6**
**Co-T (ours)**	78.1	36.9	84.0	9.3	0.2	47.4	49.2	19.3	89.0	-	89.6	77.9	52.3	91.5	-	60.3	-	47.1	64.7	**64.6**	**56.0**	**35.4**	**+20.6**
GTAV (Source) → Cityscapes																							
AdSegNet [42]	86.5	36.0	79.9	23.4	23.3	23.9	35.2	14.8	83.4	33.3	75.6	58.5	27.6	73.7	32.5	35.4	3.9	30.1	28.1	-	42.4	36.6	+5.8
IntraDA [43]	90.6	36.1	82.6	29.5	21.3	27.6	31.4	23.1	85.2	39.3	80.2	59.3	29.4	86.4	33.6	53.9	0.0	32.7	37.6	-	46.3	36.6	+9.7
CBST [11]	89.6	58.9	78.5	33.0	22.3	41.4	48.2	39.2	83.6	24.3	65.4	49.3	20.2	83.3	39.0	48.6	12.5	20.3	35.3	-	47.0	35.4	+11.6
CRST [15]	91.7	45.1	80.9	29.0	23.4	43.8	47.1	40.9	84.0	20.0	60.6	64.0	31.9	85.8	39.5	48.7	25.0	38.0	47.0	-	49.8	35.4	+14.4
DACS [21]	89.9	39.6	87.8	30.7	39.5	38.5	46.4	52.7	87.9	43.9	88.7	67.2	35.7	84.4	45.7	50.1	0.0	27.2	33.9	-	52.1	32.8	+19.3
DSP [19]	92.4	48.0	87.4	33.4	35.1	36.4	41.6	46.0	87.7	43.2	89.8	66.6	32.1	89.9	57.0	56.1	0.0	44.1	57.8	-	55.0	36.6	+18.4
MFA [22]	94.5	61.1	87.6	41.4	35.4	41.2	47.1	45.7	86.6	36.6	87.0	70.1	38.3	87.2	39.5	54.7	0.3	45.4	57.7	-	55.7	**45.6**	+10.1
RED [18]	94.4	60.9	88.0	39.4	41.8	43.2	49.0	56.0	88.0	45.8	87.7	67.5	38.0	90.0	57.6	51.9	0.0	46.5	55.2	-	57.9	34.8	+23.1
ProDA [44]	87.8	56.0	79.7	46.3	44.8	45.6	53.5	53.5	88.6	45.2	82.1	70.7	39.2	88.8	45.5	59.4	1.0	48.9	56.4	-	57.5	36.6	+20.9
**Co-T (ours)**	89.9	51.0	89.0	40.0	34.2	51.6	56.5	51.3	89.5	50.1	89.8	71.8	46.5	90.9	55.7	56.7	0.0	52.6	64.2	-	**59.5**	28.5	**+31.0**
Synscapes (Source) → Cityscapes																							
AdSegNet [42]	94.2	60.9	85.1	29.1	25.2	38.6	43.9	40.8	85.2	29.7	88.2	64.4	40.6	85.8	31.5	43.0	28.3	30.5	56.7	-	52.7	**45.3**	+7.4
IntraDA [43]	94.0	60.0	84.9	29.5	26.2	38.5	41.6	43.7	85.3	31.7	88.2	66.3	44.7	85.7	30.7	53.0	29.5	36.5	60.2	-	54.2	**45.3**	+8.9
**Co-T (ours)**	91.4	55.7	81.6	34.5	38.9	53.6	64.7	67.4	91.0	48.7	93.4	77.5	42.4	93.1	18.3	20.8	1.2	60.0	74.2	-	**58.3**	45.0	**+13.3**
GTAV + Synscapes (Source) → Cityscapes																							
MADAN [45]	94.1	61.0	86.4	43.3	32.1	40.6	49.0	44.4	87.3	47.7	89.4	61.7	36.3	87.5	35.5	45.8	31.0	33.5	52.1	-	55.7	**51.6**	+4.1
MsDACL [20]	93.6	59.6	87.1	44.9	36.7	42.1	49.9	42.5	87.7	47.6	89.9	63.5	40.3	88.2	41.0	58.3	53.1	37.9	57.7	-	59.0	**51.6**	+7.4
**Co-T (ours)**	96.3	74.7	90.4	48.8	49.1	58.3	61.5	67.0	90.7	54.7	93.5	79.4	57.7	90.4	45.6	85.1	59.9	60.4	70.9	-	**70.2**	50.0	**+20.2**

**Table 2:** Hyper-parameters of our method (Section 3 and Algorithms 1–3). Datasets: GTAV (G), Synscapes (S), SYNTHIA (SIA), Cityscapes (C), BDD100K (B), Mapillary Vistas (M).

								$M_{\mathrm{df}}$		$H_{\mathrm{st}}$				$H_{\mathrm{ct}}$				
Source	Target	N	n	Δp	$C_{m}$	$C_{M}$	$N_{\mathrm{MB}}$	$p_{\mathrm{MB}}$	$p_{\mathrm{CM}}$	$P_{m}$	$P_{M}$	$K_{m}$	$K_{M}$	$P_{m}$	$P_{M}$	K	w	λ
SIA	C	500	100	0.05	0.5	0.9	4	0.75	0.5	0.5	0.6	1	10	0.5	0.6	5	1	0.8
S, G + S	C, M	500	100	0.05	0.5	0.9	4	0.75	0.5	0.5	0.6	1	10	0.5	0.6	5	1	0.8
G	C	500	100	0.05	0.5	0.9	4	0.75	0.5	0.3	0.5	1	10	0.5	0.6	5	1	0.8
G + S	B	500	100	0.05	0.5	0.9	2	0.5	0.5	0.3	0.5	1	10	0.5	0.6	5	1	0.8

**Table 3:** Co-training results compared to baseline (Source), LAB adjustment pre-processing (SrcLAB), self-training stage, and upper-bound (SrcLAB + Target). Note that Target refers to using 100% of the target domain images labeled for training by a human oracle. CB corresponds to the class balance policy applied on GTAV. We remind also that, before running our co-training procedure (self-training stage and co-training loop), we apply target LAB adjustment on the synthetic datasets. In this table, the target domain is always Cityscapes.

Methods	Road	Sidewalk	Building	Wall	Fence	Pole	Traffic Light	Traffic Sign	Vegetation	Terrain	Sky	Person	Rider	Car	Truck	Bus	Train	Motorbike	Bike	mIoU
SYNTHIA (Source) → Cityscapes																				
Source	38.47	17.42	75.39	4.92	0.22	30.58	17.79	15.48	73.86	-	80.55	63.28	22.57	62.47	-	27.12	-	13.22	23.92	35.46
SrcLAB	58.5	22.23	78.33	6.03	0.28	37.8	17.0	15.64	78.01	-	79.36	63.05	22.48	78.91	-	32.1	-	13.31	28.8	39.48
Self-training stage (ours)	72.14	30.37	82.97	2.97	0.11	43.72	34.49	14.00	87.09	-	86.87	73.82	43.45	87.57	-	42.44	-	21.69	56.36	48.74
Co-training proce. (ours)	78.14	36.98	84.07	9.34	0.28	47.49	49.2	19.35	89.07	-	89.62	77.92	52.32	91.50	-	60.37	-	47.10	64.76	56.09
SrcLAB + Target	97.92	84.42	92.60	53.87	61.70	65.93	70.67	78.00	92.71	(65.68)	94.98	83.29	66.49	95.32	(77.37)	88.20	(71.84)	67.45	78.13	79.48
Δ(SrcLAB vs. Source)	20.03	4.81	2.94	1.11	0.06	7.22	−0.79	0.16	4.15	-	−1.19	−0.23	−0.09	16.44	-	4.98	-	0.09	4.88	4.02
Δ(Co-t vs. Source)	39.62	18.38	8.24	4.33	0.06	16.84	31.41	3.87	13.81	-	9.04	14.6	29.66	26.29	-	19.71	-	33.86	40.74	19.40
Δ(Co-t vs. SrcLAB)	19.64	14.75	5.74	3.31	0	9.69	32.2	3.71	11.06	-	10.26	14.87	29.84	12.59	-	28.27	-	33.79	35.96	16.6
Δ(Co-t vs. Self-t)	6.00	6.61	1.10	6.37	0.17	3.77	14.71	5.35	1.98	-	2.75	4.10	8.87	3.93	-	17.93	-	25.41	8.40	7.34
ΔCo-t vs. SrcLAB + Tgt)	−19.83	−48.62	−8.97	−44.62	−61.42	−18.51	−21.47	−58.65	−5.04	-	−5.39	−5.41	−14.26	−6.56	-	−41.37	-	−20.37	−13.47	−24.62
GTAV (Source) → Cityscapes																				
Source	51.85	13.57	64.71	8.19	15.86	14.39	31.66	10.86	71.95	6.91	38.21	55.65	22.21	72.40	32.62	9.76	0.0	9.93	11.14	28.52
SrcLAB	75.25	23.38	76.59	19.72	16.86	32.28	28.37	13.73	81.75	25.47	46.71	64.0	31.76	84.14	32.29	16.23	0.08	23.41	27.27	37.86
SrcLAB + CB	73.34	26.30	73.50	29.57	21.16	35.04	42.78	20.09	84.64	26.48	53.20	63.02	40.77	81.90	34.16	31.56	4.74	36.05	34.07	42.76
Self-training stage (ours)	85.31	36.82	85.11	41.09	25.62	46.39	45.19	33.44	88.98	45.55	72.99	69.54	42.43	89.36	44.42	57.5	1.28	45.51	59.78	53.49
Co-training proce. (ours)	89.92	51.03	89.09	40.05	34.23	51.61	56.54	51.36	89.50	50.12	89.83	71.88	46.50	90.91	55.72	56.77	0.0	52.61	64.21	59,57
SrcLAB + Target	98.20	85.43	92.74	59.07	63.05	65.26	69.43	77.10	92.63	65.26	94.70	82.11	63.22	95.22	85.05	86.07	67.27	64.84	77.21	78.10
Δ(SrcLAB + CB vs. Source)	21.49	12.73	8.79	21.38	5.3	20.65	11.12	9.23	12.69	19.57	14.99	7.37	18.56	9.5	1.54	21.8	4.74	26.12	22.93	14.24
Δ(Co-t vs. Source)	38.07	37.46	24.38	31.86	18.37	37.22	24.88	40.5	17.55	43.21	51.62	16.23	24.29	18.51	23.1	47.01	0.0	42.68	53.07	31.05
Δ(Co-t vs. SrcLAB + CB)	16.58	24.73	15.59	10.48	13.07	16.57	13.76	31.27	4.86	23.64	36.63	8.86	5.73	9.01	21.56	25.21	−4.74	16.56	30.14	16.81
Δ(Co-t vs. Self-t)	4.61	14.21	3.98	−1.04	8.61	5.22	11.35	17.92	0.52	4.57	16.84	2.34	4.07	1.55	11.3	−0.73	−1.28	7.1	4.43	6.08
Δ(Co-t vs. SrcLAB + Tgt)	−8.28	−34.4	−3.65	−19.02	−28.82	−13.65	−12.89	−25.74	−3.13	−15.14	−4.87	−10.23	−16.72	−4.31	−29.33	−29.3	−67.27	−12.23	−13.0	−18.53
Synscapes (Source) → Cityscapes																				
Source	83.81	42.15	61.87	26.10	21.69	44.65	47.12	53.86	81.30	33.57	53.53	67.79	29.68	85.66	14.81	6.66	2.36	34.94	63.53	45.01
SrcLAB	78.39	37.47	67.39	16.45	19.09	48.5	51.79	58.54	83.18	29.89	64.79	70.17	29.27	85.39	18.42	10.42	3.32	36.48	64.61	45.98
Self-training stage (ours)	89.55	50.19	84.26	33.61	37.67	57.29	60.11	64.00	90.61	47.13	91.22	72.15	21.17	91.99	15.38	20.09	9.35	44.94	70.78	55.34
Co-training proce. (ours)	91.46	55.76	81.63	34.58	38.92	53.66	64.74	67.43	91.02	48.72	93.45	77.54	42.40	93.14	18.35	20.84	1.29	60.03	74.22	58.38
SrcLAB + Target	98.03	84.49	92.90	59.10	63.70	67.18	71.67	79.50	92.74	65.51	94.81	83.93	68.07	95.45	82.89	91.83	83.79	70.91	79.24	80.30
Δ(SrcLAB vs. Source)	0.97	−5.42	−4.68	5.52	−9.65	−2.6	3.85	4.67	4.68	1.88	−3.68	11.26	2.38	−0.41	−0.27	3.61	3.76	0.96	1.54	1.08
Δ(Co-t vs. Source)	7.65	13.61	19.76	8.48	17.23	9.01	17.62	13.57	9.72	15.15	39.92	9.75	12.72	7.48	3.54	14.18	−1.07	25.09	10.69	13.37
Δ(Co-t vs. SrcLAB)	13.07	18.29	14.24	18.13	19.83	5.16	12.95	8.89	7.84	18.83	28.66	7.37	13.13	7.75	−0.07	10.42	−2.03	23.55	9.61	12.40
Δ(Co-t vs. Self-t)	1.91	5.57	−2.63	0.97	1.25	−3.63	4.63	3.43	0.41	1.59	2.23	5.39	21.23	1.15	2.97	0.75	−8.06	15.09	3.44	3.04
Δ(Co-t vs. SrcLAB + Tgt)	−6.57	−28.73	−11.27	−24.52	−24.78	−13.52	−6.93	−12.07	−1.72	−16.79	−1.36	−6.39	−25.67	−2.31	−64.54	−70.99	−82.5	−10.88	−5.02	−21.92
GTAV + Synscapes (Source) → Cityscapes																				
Source	66.39	33.54	79.58	29.43	40.24	49.73	56.12	46.51	81.22	18.40	79.06	73.18	29.67	85.25	43.00	6.46	23.02	47.71	61.63	50.01
SrcLAB	87.97	47.45	85.14	34.31	43.16	49.82	57.16	47.85	88.88	45.00	82.53	72.58	38.22	89.16	51.91	61.31	40.06	43.64	60.85	59.32
Self-training stage (ours)	93.93	66.08	89.95	46.40	48.13	56.30	59.65	65.16	90.25	52.22	93.33	75.97	41.15	90.40	44.98	75.08	65.52	55.52	71.98	67.47
Co-training proce. (ours)	96.30	74.72	90.44	48.89	49.15	58.36	61.52	67.05	90.75	54.75	93.52	79.48	57.71	90.48	45.61	85.11	59.95	60.41	70.96	70.23
SrcLAB + Target	97.88	83.43	92.33	64.15	61.77	63.45	68.04	75.17	92.31	62.74	94.08	82.00	64.16	95.01	84.25	89.66	75.56	63.28	76.07	78.18
Δ(SrcLAB vs. Source)	21.58	13.91	5.56	4.88	2.92	0.09	1.04	1.34	7.66	26.6	3.47	−0.6	8.55	3.91	8.91	54.85	17.04	−4.07	−0.78	9.31
Δ(Co−t vs. Source)	29.91	41.18	10.86	19.46	8.91	8.63	5.40	20.54	9.53	36.35	14.46	6.3	28.04	5.23	2.61	78.65	36.93	12.70	9.33	20.22
Δ(Co-t vs. SrcLAB)	8.33	27.27	5.30	14.58	5.99	8.54	4.36	19.20	1.87	9.75	10.99	6.9	19.49	1.32	−6.3	23.8	19.89	16.77	9.28	10.91
Δ(Co-t vs. Self-t)	2.37	8.64	0.49	2.49	1.02	2.06	1.87	1.89	0.5	2.53	0.19	3.51	16.56	0.08	0.63	10.03	−5.57	4.89	−1.85	2.76
Δ(Co-t vs. SrcLAB + Tgt)	−1.58	−8.71	−1.89	−15.26	−12.62	−5.09	−6.52	−8.12	−1.56	−7.99	−0.56	−2.52	−6.45	−4.53	−38.64	−4.55	−15.61	−2.87	−5.94	−7.95

**Table 4:** Contribution of the main components of our proposal. Case study: GTAV + Synscapes → Cityscapes.

			Co-Training Procedure			
			Self-Training Stage			
	Baseline	+LAB	+MixBatch	+ClassMix	+Co-Training Loop	Upper Bound
mIoU	50.01	59.32	66.18	67.47	70.23	78.18
Gain	-	+9.31	+6.86	+1.29	+2.76	-

**Table 5:** Analogous to Table 3 with BDD100K and Mapillary as target domains.

Methods	Road	Sidewalk	Building	Wall	Fence	Pole	Traffic Light	Traffic Sign	Vegetation	Terrain	Sky	Person	Rider	Car	Truck	Bus	Train	Motorbike	Bike	mIoU
GTAV + Synscapes (Source) → BDD100K																				
Source	67.83	20.84	54.86	9.00	27.57	30.24	31.74	20.75	62.69	15.39	63.75	54.53	24.08	65.92	12.82	9.10	0.07	39.58	39.04	34.20
SrcLAB	74.22	26.07	68.48	7.94	15.51	31.09	38.69	22.90	69.33	25.92	74.27	59.35	18.81	72.79	23.66	19.75	0.02	54.72	35.48	38.68
Self-training stage (ours)	88.52	26.21	78.77	14.48	35.41	41.40	49.27	31.74	75.86	35.89	88.85	60.39	35.22	85.48	35.04	42.29	0.00	51.28	47.40	48.60
Co-training proce. (ours)	88.43	31.63	80.05	13.05	39.89	41.81	46.12	29.67	76.06	37.79	89.50	63.08	39.94	85.78	40.52	42.71	0.07	53.60	50.40	50.11
SrcLAB + Target	93.33	60.89	84.41	31.45	47.74	49.62	55.33	47.77	85.00	42.77	92.60	66.10	38.91	88.11	40.63	71.09	0.00	57.71	54.90	58.33
Δ(SrcLAB vs. Source)	6.39	5.23	9.62	−1.06	−12.06	0.85	6.95	2.15	6.64	10.53	10.52	4.82	−5.27	6.87	10.84	10.65	−0.05	15.14	−3.56	4.48
Δ(Co-t vs. Source)	25.5	40.05	29.55	22.45	20.17	19.38	23.59	27.02	22.31	27.38	28.85	11.57	14.83	22.19	27.81	61.99	−0.07	18.13	15.86	24.13
Δ(Co-t vs. SrcLAB)	19.11	34.82	19.93	23.51	32.23	18.53	16.64	24.87	15.67	16.85	18.33	6.75	20.1	15.32	16.97	51.34	−0.02	2.99	19.42	19.65
Δ(Co-t vs. Self-t)	−0.09	5.42	1.28	−1.43	4.48	0.41	−3.15	−2.07	0.2	1.9	0.65	2.69	4.72	0.3	5.48	0.42	0.07	2.32	3.0	1.51
Δ(Co-t vs. SrcLAB + Tgt)	−4.9	−29.26	−4.36	−18.4	−7.85	−7.81	−9.21	−18.1	−8.94	−4.98	−3.1	−3.02	1.03	−2.33	−0.11	−28.38	0.07	−4.11	−4.5	−8.22
GTAV + Synscapes (Source) → Mapillary Vistas																				
Source	68.81	31.73	68.88	25.20	37.94	38.79	49.79	20.57	73.27	29.66	80.62	63.81	42.75	80.65	35.74	16.86	1.85	44.56	47.09	45.19
SrcLAB	72.62	43.18	70.89	17.21	25.18	35.05	57.74	55.73	76.78	27.09	88.72	71.34	24.34	77.89	46.29	47.37	0.00	34.27	46.77	48.34
Self-training stage (ours)	89.44	53.30	85.28	36.57	44.89	47.10	59.18	65.94	84.58	48.25	97.44	74.23	55.71	89.37	58.34	59.45	1.47	49.44	51.50	60.60
Co-training proce. (ours)	90.44	57.83	85.59	36.38	45.56	49.64	59.73	67.62	84.27	47.08	96.79	74.80	56.05	90.42	56.34	49.86	10.71	49.62	55.94	61.30
SrcLAB + Target	94.02	69.46	88.70	51.38	60.17	57.59	64.21	75.16	90.70	69.35	98.27	76.02	56.70	91.42	60.49	73.35	33.81	60.87	66.63	70.44
Δ(SrcLAB vs. Source)	−2.93	−0.24	0.73	−8.10	−8.64	5.25	5.94	21.32	3.34	2.73	1.74	3.47	4.75	1.86	5.84	−3.15	1.09	7.51	6.69	2.59
Δ(Co-t vs. Source)	21.63	26.10	16.71	11.18	7.62	10.85	9.94	47.05	11.0	17.42	16.17	10.99	13.30	9.77	20.60	33.0	8.86	5.06	8.85	16.11
Δ(Co-t vs. SrcLAB)	24.56	26.34	15.98	19.28	16.26	5.6	4.0	25.73	7.66	14.69	14.43	7.52	8.55	7.91	14.76	36.15	7.77	−2.45	2.16	13.52
Δ(Co-t vs. Self-t)	1.0	4.53	0.31	−0.19	0.67	2.54	0.55	1.68	−0.31	−1.17	−0.65	0.57	0.34	1.05	−2.0	−9.59	9.24	0.18	4.44	0.7
Δ(Co-t vs. SrcLAB + Tgt)	−3.58	−11.63	−3.11	−15.0	−14.61	−7.95	−4.48	−7.54	−6.43	−22.27	−1.48	−1.22	−0.65	−1.0	−4.15	−23.49	−23.1	−11.25	−10.69	−9.14

**Figure A1:**
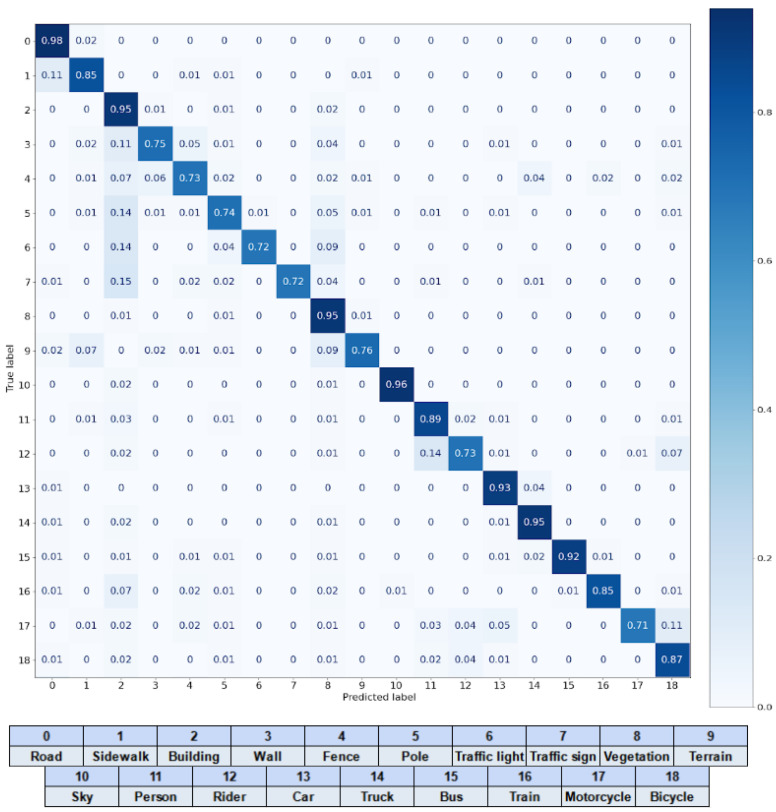
Confusion matrix of the co-training model trained with GTAV + Synscapes as source data and Cityscapes as target data.

**Figure A2:**
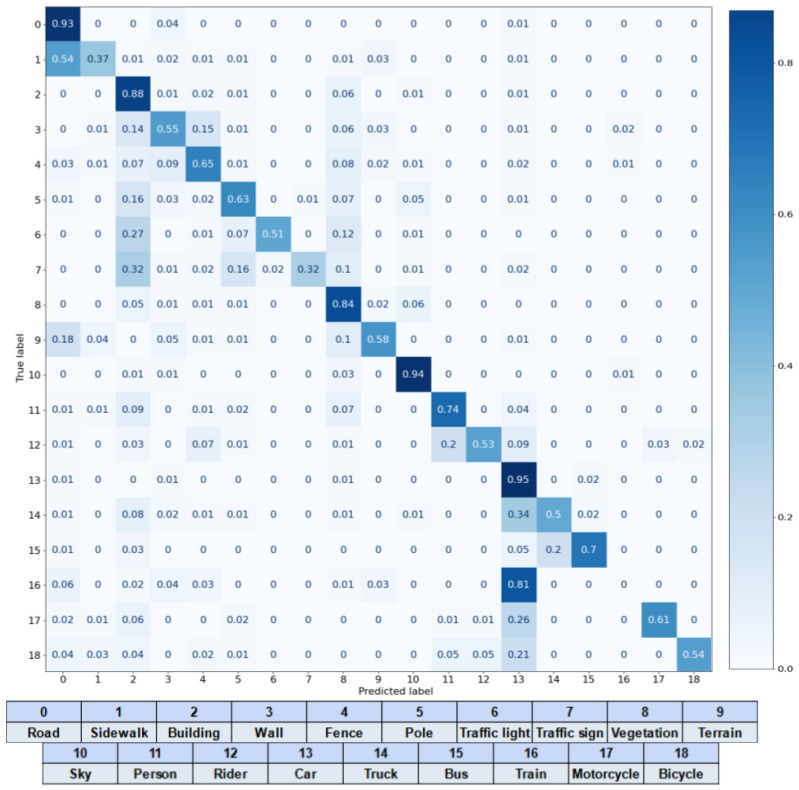
Confusion matrix of the co-training model trained with GTAV + Synscapes as source data and BDD100K as target data.

**Figure A3:**
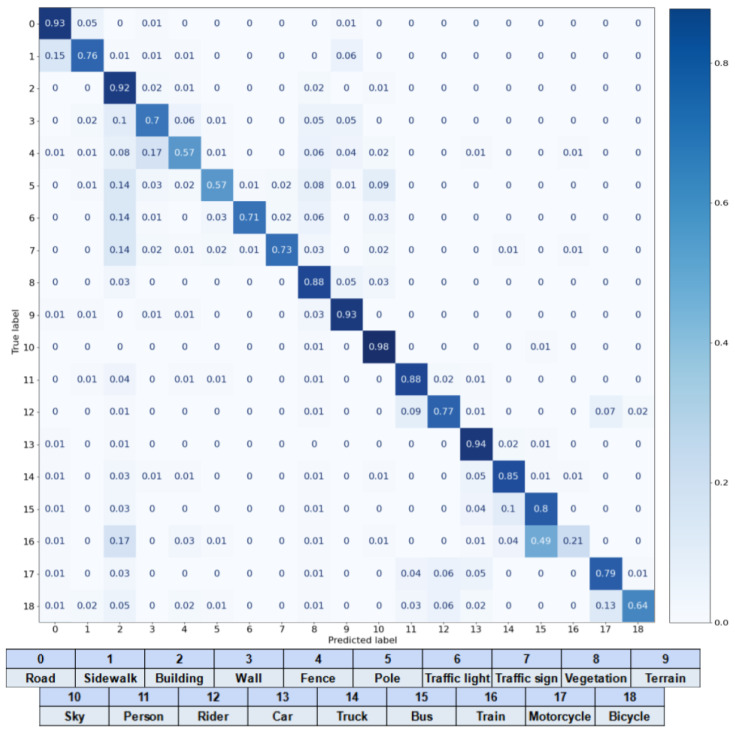
Confusion matrix of the co-training model trained with GTAV + Synscapes as source data and Mapillary Vistas as target data.

**Figure A4:**
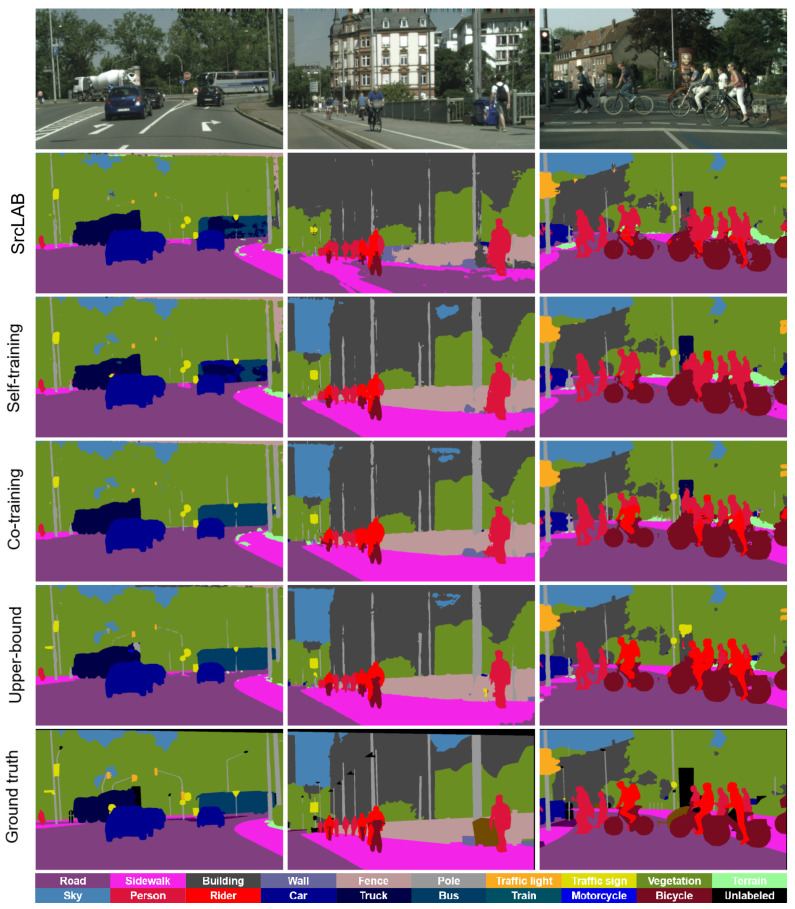
Qualitative results on the validation set of Cityscapes when relying on GTAV + Synscapes as source data. The baseline model is trained using only these source data, the upper-bound model uses these source data and all the labeled training data of Cityscapes. Self-training and co-training models rely on the source data and the same training data from Cityscapes but without the labeling information.

**Figure A5:**
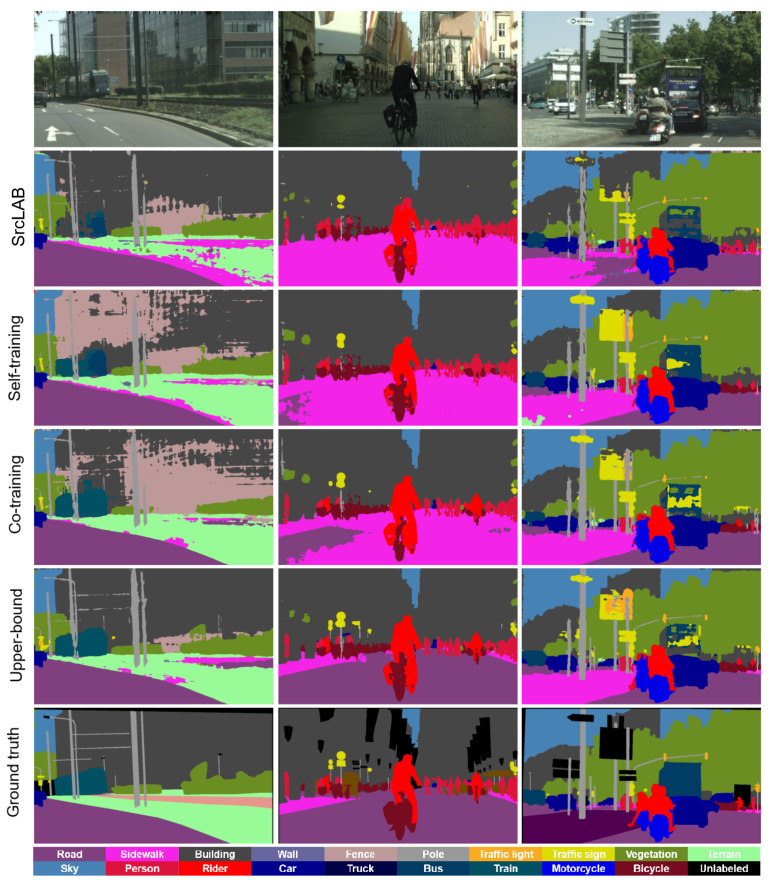
Analogous to Figure A4, focusing on problematic examples.

**Figure A6:**
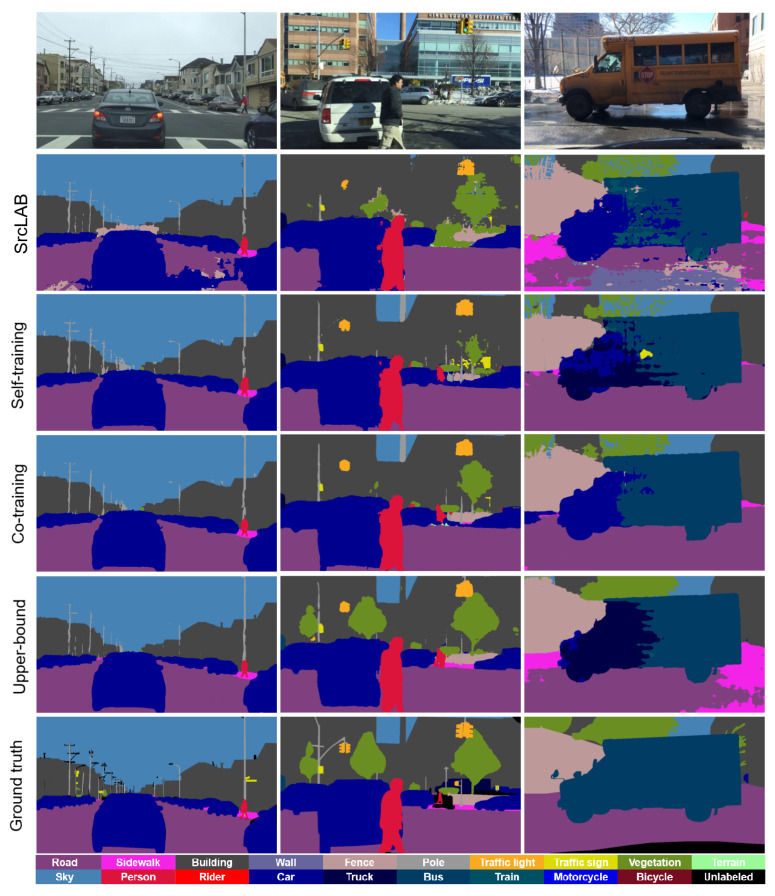
Qualitative results similar to Figure A4 when using BDD100K as target.

**Figure A7:**
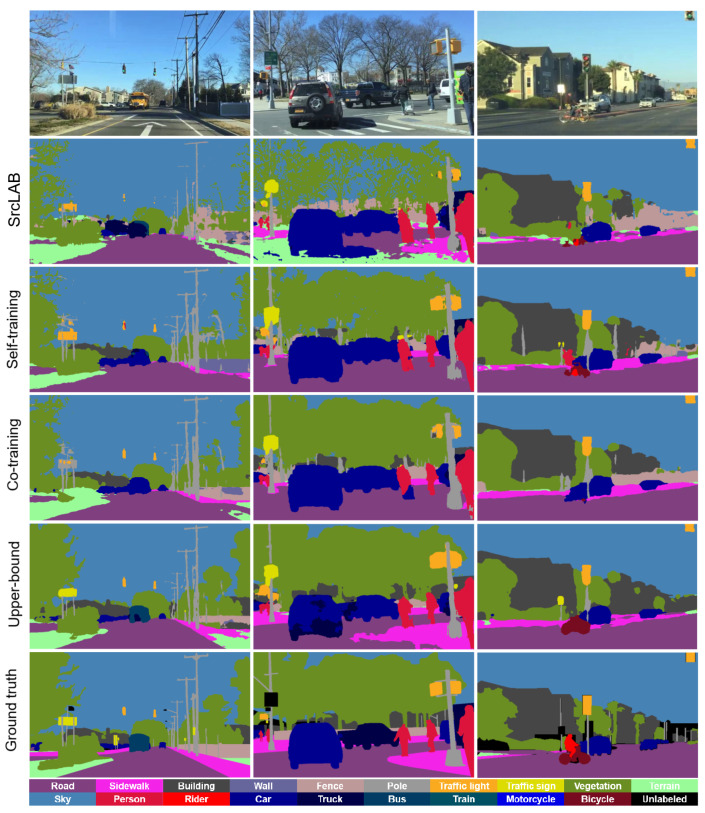
Analogous to Figure A6, focusing on problematic examples.

**Figure A8:**
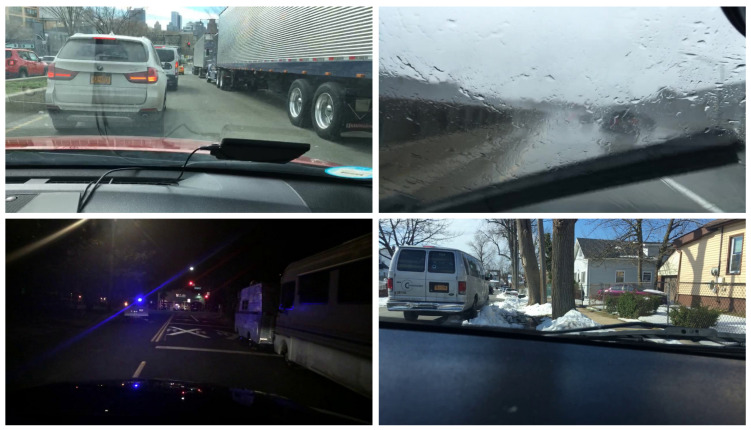
Image samples from BDD100K dataset.

**Figure A9:**
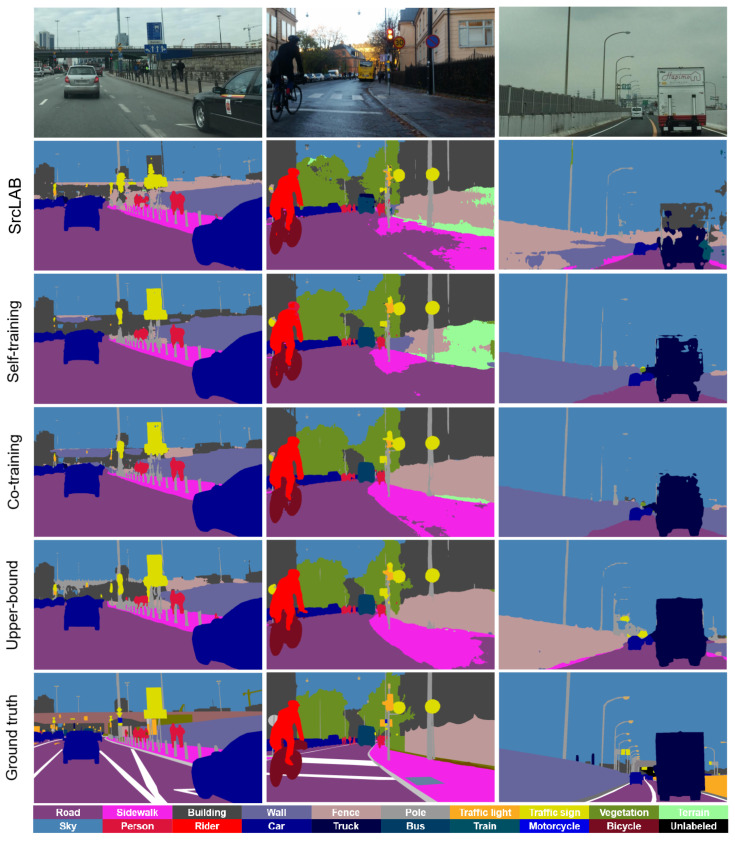
Qualitative results similar to Figure A4 when using Mapillary Vistas as target.

**Figure A10:**
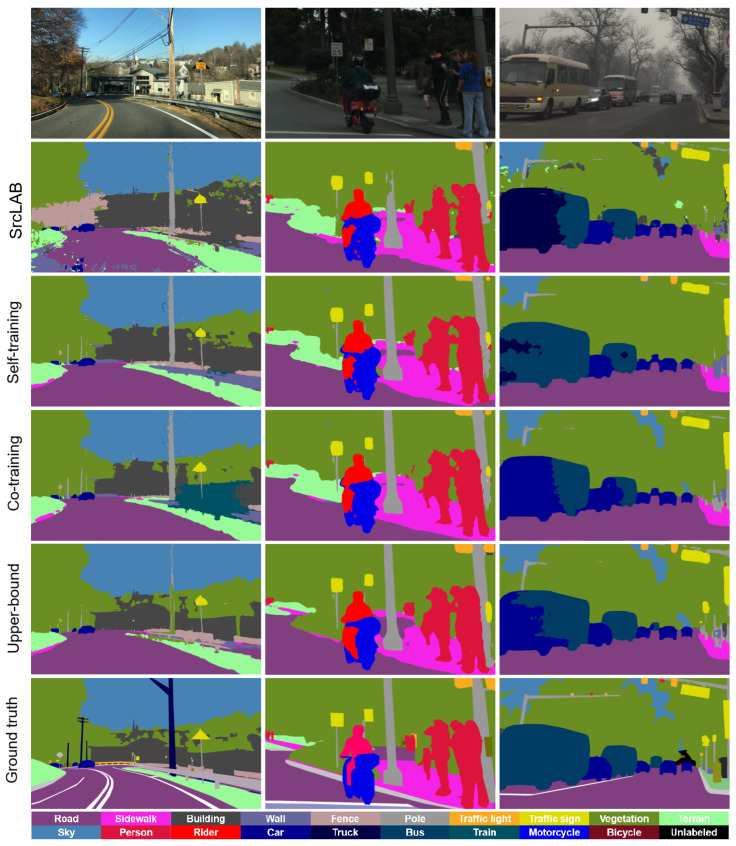
Analogous to Figure A9, focusing on problematic examples.

## Data Availability

All datasets used in this study have been downloaded from well-known publicly available sources, whose associated papers are properly cited and so included in the references.

## Appendix A. Additional Datasets Information

Table A1 presents statistics about the content of the different datasets. Focusing on the synthetic datasets, we observe that GTAV has few cases of Bicycle and Train, thus explaining why the semantic segmentation baseline performs poorly on these classes. On other hand, SYNTHIA and Synscapes are overall well-balanced, however, in Synscapes, the examples of Bus, Train, and Truck are very similar in shape. On other hand, SYNTHIA is less photo-realistic than Synscapes.

**Table A1:** Content statistics for the considered datasets. For each class, we indicate the percentage (%) of: (1) Images containing samples of the class, and (2) Pixels in the dataset with the class label.

		Road	Sidewalk	Building	Wall	Fence	Pole	Traffic Light	Traffic Sign	Vegetation	Terrain	Sky	Person	Rider	Car	Truck	Bus	Train	Motorbike	Bicycle
SYNTHIA	Images	99.9	99.9	99.9	41.7	79.9	99.9	68.1	94.3	97.3	0.0	93.9	99.9	99.5	95.8	0.0	76.4	0.0	84.1	99.5
	Pixels	18.54	19.31	29.43	0.27	0.26	1.04	0.04	0.10	10.15	0.0	6.80	4.25	0.47	4.04	0.0	1.53	0.0	0.21	0.22
GTAV	Images	99.3	97.3	99.8	95.7	84.2	99.2	74.3	60.5	99.3	97.8	99.2	93.9	13.1	91.7	67.0	20.4	4.5	16.7	1.8
	Pixels	32.1	8.29	16.91	1.85	0.63	1.06	0.13	0.08	7.6	2.14	13.53	0.36	0.03	2.51	1.13	0.37	0.06	0.03	0.1
Synscapes	Images	99.9	98.7	99.1	32.8	98.2	99.6	96.7	98.9	97.6	48.2	98.0	99.7	82.3	98.5	86.9	80.8	65.8	86.5	89.5
	Pixels	28.29	6.82	22.57	1.13	1.37	2.07	0.37	0.53	13.93	0.88	8.31	3.18	0.79	5.59	0.79	1.08	1.17	0.6	0.52
Cityscapes	Images	98.6	94.5	98.6	32.6	43.6	99.1	55.7	94.4	70.3	55.6	90.3	78.8	34.4	95.2	12.1	9.2	4.8	17.2	55.3
	Pixels	32.63	5.39	20.19	0.58	0.78	1.09	0.18	0.49	14.08	1.03	3.55	1.08	0.12	6.19	0.24	0.21	0.21	0.09	0.37
BDD100K	Images	96.5	66.7	88.4	15.4	30.6	95.0	47.1	75.3	91.7	36.7	94.8	34.7	5.2	97.3	30.5	15.0	0.7	3.8	6.4
	Pixels	21.26	2.03	13.24	0.48	1.03	0.94	0.18	0.34	13.2	1.03	17.26	0.25	0.02	8.13	0.97	0.56	0.01	0.02	0.05
Mapillary	Images	98.8	72.2	91.3	46.2	62.7	98.6	51.0	91.0	96.8	46.9	98.9	50.5	19.0	93.4	24.0	15.8	1.3	15.1	16.7
	Pixels	19.42	2.99	12.47	0.75	1.26	0.9	0.18	0.45	14.94	1.05	29.33	0.31	0.06	3.36	0.37	0.26	0.02	0.06	0.07

## Appendix B. Additional Co-Training Information

Figure A1 shows the confusion matrix of the semantic segmentation model trained with the pseudo-labels provided by co-training on GTAV + Synscapes (source) and Cityscapes (target). We can see how background classes such as Road, Building, Vegetation, and Sky reach a ∼95% precision, where ’∼’ means *approximately*. All classes corresponding to dynamic objects show a ∼90% precision, except for Motorbike and Rider with ∼70%. Riders may be confused with pedestrians, and motorbikes with bicycles. This problem could be addressed by injecting more Rider samples in the source data, including corner cases where they appear with pedestrians around. In addition, having more synthetic samples showing motorbikes and bicycles may help to better differentiate such classes.

Figure A2 is analogous to Figure A1 but for BDD100K as target data. Again, most classes corresponding to environmental elements (Road, Building, Vegetation, and Sky) have high precision. However, some of them have a large margin for improvement. For instance, the Sidewalk class tends to be labeled as Road, which we think is due to lacking real-world images with sidewalks; in Table A1, we can see that only the ∼66% of the training images contain sidewalks, while this statistics reaches the ∼98% in the case of Cityscapes. Wall, Fence, and Pole classes form a sort of confusion set. Sometimes, pixels of these classes are also labeled as Vegetation because their instances occlude instances of Wall/Fence/Pole or vice versa. Traffic lights and signs are frequently labeled as Building/Pole/Vegetation. Here, a different labeling policy may also be introducing confusion on the trained model. While the rear part of traffic lights and signs is not labeled in Synscapes and Cityscapes, they are in BDD100K. Other classes such as Truck, Motorbike, and Bike, tend to be labeled as Car. The Truck class is also under a discrepancy in labeling policy, since pick-up vehicles are labeled as Truck in BDD100K but as Car in the others datasets.

Figure A3 is analogous to Figure A1 but for Mapillary Vistas as target data. The diagonal scores and confusion cases are similar to those of Cityscapes. We can observe cases of high precision but not so high IoU. For instance, ∼93% and ∼47% for the class Terrain, respectively. Other classes showing a similar pattern are Truck, Bus, and Motorbike. We think this can be at least partially due to differences in labeling policies. Mapillary Vistas accounts for around one hundred different classes, which cannot be easily mapped to the 19 classes of Cityscapes. Then, using only 19 classes while setting as unlabeled the rest, drives to a ground truth with less information per training image than in cases such as Cityscapes and BDD100K.

## Appendix C. Additional Qualitative Analysis

**GTAV + Synscapes → Cityscapes**: In Figure A4 and Figure A5 we show additional qualitative results obtained on Cityscapes, when using GTAV + Synscapes as source domain.

Figure A4 remarks where several improvements from the co-training model vs. self-training one appear. In the example in the left column, we see that the baseline and self-training models have problems labeling a bus while the co-training model labels all the dynamic objects accurately as the upper-bound model does. In the mid column, the co-training model improves the labeling of the sidewalk, the closest person and rider. The last column shows a challenging case where several pedestrians mixed with cyclists are crossing the road. The baseline and self-training models are poor at distinguishing riders from pedestrians. The co-training model is able to improve on classifying riders over the baseline and self-training models, but not reaching the performance of the upper-bound model in this case.

Figure A5 shows examples of wrong labeling even from the co-training model. The left column shows a building erroneously labeled by all models except by the upper-bound one. A large area of the building is labeled as Fence, which we believe is due to the reflections seen in the facade windows. Furthermore, the variability of buildings in the synthetic data is not enough to cover these variants seen in real-world scenarios. The upper-bound model properly labels most of the building, but still labels part of its bottom as Fence. The mid column shows a usual troublesome case in Cityscapes, where a stone-based road is labeled as Sidewalk. Note that stones are also used to build sidewalks. The right column shows a bus with some kind of advertising on the back, which induces all the models (including the upper-bound one) to label the bus as a mixture of Traffic Sign and Building. We note that, overall, there are not sufficient training samples of this type.

**GTAV + Synscapes → BDD100K**: In Figure A6 and Figure A7 we present qualitative results changing the target to BDD100K.

Figure A6 shows how noisy the baseline model is in this case. This is due to variability regarding weather conditions, lighting, and on-board cameras. Note that, contrarily to the case of Cityscapes, BDD100K cameras are not even installed in the same position from car to car, not even in the same car model in all the cases (see Figure A8). In the left column of Figure A6, we see how both self-training and co-training models clearly perform much better than the baseline, in fact, similarly to the upper-bound one. In the mid column, the co-training model is the one performing most similarly to the upper bound. In the last column, self-training, co-training, and upper-bound models have problems labeling the bus cabin, which is confused with a truck cabin (upper-bound), a car cabin (co-training), and a bit of both (self-training as coming from the baseline).

Figure A7 shows examples of wrong labeling even from the co-training model. The left column shows an example where a far bus is confused with a Truck (baseline) or a Car (self-training and co-training), and the sidewalk is largely confused with Terrain/Road (baseline and co-training), Road (self-training), even the upper-bound confuses the part of the sidewalk with Terrain. Traffic lights are also misclassified. The mid column also shows failures in sidewalk classification for self-training and co-training models, although both label the road better than the baseline. These models label the closest car even better than the upper-bound model. The right column shows an extreme case where all models have difficulties labeling a case of rider-with-bicycle. The baseline model provides some insufficient cues, self-training improves them, but the co-training model labels the rider-with-bicycle partially as Car and partially as Road. Even the upper-bound model misses the rider, only properly labeling the bicycle.

**GTAV + Synscapes → Mapillary**: In Figure A9 and Figure A10 we introduce qualitative results changing the target to Mapillary Vistas.

Figure A9 aims to show cases where the co-training model performs similarly or even better than the upper-bound one. In the left column, the co-training model performs better than the rest of the models by avoiding parts of the wall being labeled as Fence. In the mid column, only the co-training and the upper-bound models perform relatively well labeling the sidewalk, the co-training model even better. In the right column, only the co-training model is properly labeling the wall and relatively well the close truck. Note how the upper-bound model labels the wall as Fence.

Figure A10 shows examples of wrong labeling from the co-training model. In the left column, some buildings are labeled as Train. In fact, these buildings have a shape and are arranged in a way that resembles train wagons. In the mid column, we see that the co-training model is labeling some vegetation as Terrain, performing a bit worse than the self-training model. In the right column, the co-training model partially labels two buses as Car, while the self-training model performs better labeling in these cases.
